# Effect of a Single High-Dose Vitamin D_3_ on the Length of Hospital Stay of Severely 25-Hydroxyvitamin D-Deficient Patients with COVID-19

**DOI:** 10.6061/clinics/2021/e3549

**Published:** 2021-11-17

**Authors:** Igor H. Murai, Alan L. Fernandes, Leila Antonangelo, Bruno Gualano, Rosa Maria Rodrigues Pereira

**Affiliations:** I Divisao de Reumatologia, Faculdade de Medicina FMUSP, Universidade de Sao Paulo, Sao Paulo, SP, BR.; II Divisao de Patologia Clinica, Hospital das Clinicas HCFMUSP, Faculdade de Medicina, Universidade de Sao Paulo, Sao Paulo, SP, BR.

**Keywords:** SARS-CoV-2, Immune System, Pneumonia, Acute Respiratory Disease

## Abstract

**OBJECTIVES:**

In this ancillary analysis of a multicenter, double-blinded, randomized, placebo-controlled trial, we investigated the effect of a single high dose of vitamin D_3_ on the length of hospital stay of patients with severe 25-hydroxyvitamin D deficiency and COVID-19.

**METHODS:**

The primary outcome was length of hospital stay, defined as the total number of days that patients remained hospitalized from the date of randomization until the date of hospital discharge. Secondary outcomes included serum levels of 25-hydroxyvitamin D, mortality during hospitalization, number of patients admitted to the intensive care unit, and number of patients who required mechanical ventilation. ClinicalTrials.gov: NCT04449718.

**RESULTS:**

Thirty-two patients were included in the study. The mean (SD) age was 58.5 (15.6) years, body mass index was 30.8 (8.6) kg/m^2^, and 25-hydroxyvitamin D level was 7.8 (1.6) ng/mL. No significant difference was observed in the median interquartile range of length of hospital stay between the vitamin D_3_ group (6.0 [4.0-18.0] days) *versus* placebo (9.5 [6.3-15.5] days) (log-rank *p*=0.74; hazard ratio, 1.13 [95% confidence interval (CI), 0.53-2.40]; *p*=0.76). Vitamin D_3_ significantly increased serum 25-hydroxyvitamin D levels in the vitamin D_3_ group compared with that in the placebo group (between-group difference, 23.9 ng/mL [95% CI, 17.7-30.1]; *p*<0.001).

**CONCLUSIONS:**

A dose of 200.000 IU of vitamin D_3_ did not significantly reduce the length of hospital stay of patients with severe 25-hydroxyvitamin D deficiency and COVID-19.

## INTRODUCTION

Vitamin D has potent antimicrobial effects, which may modulate the immune system (1) and protect against respiratory diseases (2). Hospitalized patients with COVID-19 may present with low levels of 25-hydroxyvitamin D [25(OH)D] (3,4). However, the role of vitamin D in the management of COVID-19 remains controversial (3,5,6). We recently showed that a single high dose of vitamin D_3_ *versus* placebo did not significantly reduce the length of hospital stay among hospitalized patients with moderate to severe COVID-19 and either normal (>30 ng/mL) or reduced levels of 25(OH)D (<20 ng/mL) (7). However, in a subsequent cohort study, we observed that COVID-19 patients with 25(OH)D levels <10 ng/mL showed a trend (*p*=0.057) of longer length of hospital stay than those with 25(OH)D levels ≥10 ng/mL (95% confidence interval [CI]: 6.4-11.6 days *versus* 6.6-7.4 days) (8).

Herein, we report on an ancillary analysis of our randomized clinical trial (7) to investigate whether, in a subset of severely 25(OH)D-deficient patients with moderate to severe COVID-19, a single high dose of vitamin D_3_ could reduce the length of hospital stay and improve other clinical outcomes.

## MATERIAL AND METHODS

In this ancillary analysis of a multicenter, double-blinded, randomized, placebo-controlled trial of a single dose of 200.000 IU of vitamin D_3_ *versus* placebo (ClinicalTrials.gov Identifier: NCT04449718), we assessed a subset of hospitalized patients with moderate to severe COVID-19 presenting with severe 25(OH)D deficiency [<10 ng/mL (9) at baseline]. Participants were enrolled from the Hospital das Clínicas HCFMUSP, Faculdade de Medicina, Universidade de São Paulo (a quaternary referral teaching hospital) and from the Ibirapuera Field Hospital, both located in São Paulo, Brazil. All patients were diagnosed with COVID-19 via polymerase chain reaction testing at the time of enrollment or using a serological assay (ELISA) to detect IgG against severe acute respiratory syndrome coronavirus 2 (SARS-CoV-2). The primary outcome was length of hospital stay, defined as the total number of days that patients remained hospitalized from the date of randomization until the date of hospital discharge. Secondary outcomes included serum levels of 25(OH)D, mortality during hospitalization, number of patients admitted to the intensive care unit, and number of patients who required mechanical ventilation.

The protocol followed the Declaration of Helsinki and local regulations and was approved by the National and Institutional Ethical Committee of the Hospital das Clínicas HCFMUSP, Faculdade de Medicina, Universidade de São Paulo, São Paulo, SP, Brazil. Written informed consent was obtained from each participant prior to enrollment. This manuscript has been reported according to the CONSORT guidelines. Further details on patient recruitment, supplementation protocol and blindness, procedures, and outcomes can be found elsewhere (7).

The log-rank test was used to compare the Kaplan-Meier estimate curves for length of hospital stay, with deaths being right-censored in the analysis. A Cox regression model was used to estimate the hazard ratio (HR) with corresponding two-sided 95% CIs. Generalized estimating equations for repeated measures were used to test possible differences in 25(OH)D levels. Statistical analyses were performed using the IBM-SPSS software, version 20.0. The significance level was set at two-sided α=0.05.

## RESULTS

Of the 237 patients who participated in the randomized controlled trial (7), 32 had severe 25(OH)D deficiency (16 in each arm). The mean (SD) age was 58.5 (15.6) years, body mass index was 30.8 (8.6) kg/m^2^, and 25(OH)D level was 7.8 (1.6) ng/mL (Table 1).

**Figure 1:**
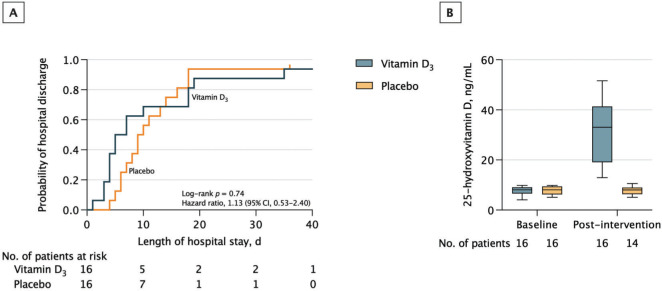
Hospital discharge and serum 25-hydroxyvitamin D levels. A, Vertical bars represent single censored events. The median (interquartile range [IQR]) observation time was not significantly different between the vitamin D_3_ group (6.0 [4.0-18.0] days) and the placebo group (9.5 [6.3-15.5] days) (log-rank *p*=0.74; HR for hospital discharge, 1.13 (95% confidence interval [CI], 0.53-2.40; *p*=0.76). B, 25-hydroxyvitamin D levels measured on the day of randomization (baseline) and on hospital discharge (post-intervention). A single high dose of vitamin D_3_ significantly increased 25-hydroxyvitamin D levels compared with the placebo (difference, 23.9 ng/mL [95% CI, 17.7-30.1]; *p*<0.001). The median IQR observation time of the post-intervention period was 6.0 (4.0-18.0) days for the vitamin D_3_ group and 9.5 (6.3-15.5) days for the placebo group. Intention-to-treat analysis was used.

There was no significant difference in the median (interquartile range [IQR]) length of hospital stay between the vitamin D_3_ group (6.0 [4.0-18.0] days) *versus* placebo (9.5 [6.3-15.5] days) (log-rank *p*=0.74; HR for hospital discharge, 1.13 [95% CI, 0.53-2.40]; *p*=0.76) (Figure 1). Importantly, the number of patients with a length of hospital stay <7 [the median time observed in the entire cohort of patients (7)] was eight in the vitamin D_3_ group and four in the placebo group (Fischer’s exact test: *p*=0.273).

A single high dose of vitamin D_3_ significantly increased the mean [SD] serum 25(OH)D levels in the vitamin D_3_ group (baseline: 7.7 [1.6] ng/mL; post: 31.7 [12.3] ng/mL) *versus* placebo (baseline: 7.9 [1.6] ng/mL; post: 7.8 [1.7] ng/mL) (between-group difference at post-intervention, 23.9 ng/mL [95% CI, 17.7-30.1]; *p*<0.001) (Figure 1).

Two patients in the vitamin D_3_ group (12.5%) and four patients in the placebo group (25.0%) were admitted to the intensive care unit during follow-up (between-group difference, -12.5% [95% CI, -39.2-14.2%]; *p*=0.65). None of the patients in the vitamin D_3_ group required mechanical ventilation *versus* one patient (6.3%) in the placebo group (*p*>0.99). There was no in-hospital mortality in the vitamin D_3_ group *versus* one death (6.3%) in the placebo group (*p*>0.99).

## DISCUSSION

In this ancillary analysis including a subset of patients with moderate to severe COVID-19 and severe 25(OH)D deficiency, we showed that a single high dose of 200.000 IU of vitamin D_3_ resulted in an approximate four-fold increase in 25(OH)D levels but did not significantly reduce length of hospital stay, mortality, admission to intensive care unit, mechanical ventilation requirement, or other clinical outcomes. Despite the well-recognized role of vitamin D in the immune system (1), findings from observational studies are controversial concerning the association between vitamin D deficiency and COVID-19 severity (3,5). In addition, a recent systematic review including randomized controlled trials did not collate sufficient evidence to conclude that vitamin D supplementation benefits COVID-19 patients (10). In the current study, the wide CIs for HR regarding length of hospital stay suggest that some patients may have benefited from the intervention, a hypothesis that needs to be tested by larger clinical trials involving severely 25(OH)D-deficient patients.

The strengths of this study include its randomized, controlled, double-blinded design, confirmation of the ability of the supplementation protocol to raise 25(OH)D levels, and assessment of patients before vaccination, which could be an important confounder affecting the clinical outcomes.

The limitations of this study were the small sample size, considering that this trial was not planned to evaluate severely 25(OH)D-deficient patients only, and the long time that elapsed from symptom onset to vitamin D_3_ administration (*i.e.*, 8.6 days), which could mask potential early benefits evoked by this intervention.

## CONCLUSION

A dose of 200.000 IU of vitamin D_3_ did not significantly reduce the length of hospital stay of hospitalized patients with COVID-19 presenting with severe 25(OH)D deficiency, although there was great heterogeneity in the responses likely associated with the low sample size. Further trials are warranted to test the efficacy of vitamin D_3_ supplementation, particularly as a pre- or post-exposure prophylaxis strategy, in patients with COVID-19 and severe 25(OH)D deficiency.

## AUTHOR CONTRIBUTIONS

Pereira RMR had full access to all the data in the study and took responsibility for the integrity of the data and the accuracy of the data analysis, supervised the study, and obtained funding. Antonangelo L was responsible for the administrative, technical and material support. Murai IH, Fernandes AL, Gualano B and Pereira RMR were responsible for the manuscript conception and design. Murai IH, Gualano B and Pereira RMR were responsible for the manuscript drafting. Murai IH and Pereira RMR were responsible for the statistical analysis. All of the authors were responsible for the data acquisition, analysis and interpretation and critical revision of the manuscript for important intellectual content.

## Figures and Tables

**Table 1:** Baseline demographic and clinical characteristics.

Characteristics	Vitamin D_3_ group (n=16)	Placebo group (n=16)
Age, mean (SD), y	55.7 (16.6)	61.3 (14.4)
Sex, No. (%)		
Male	9 (56.3)	6 (37.5)
Female	7 (43.8)	10 (62.5)
Race, No. (%)		
White	5 (31.3)	10 (62.5)
Pardo^a^	9 (56.3)	3 (18.8)
Black	2 (12.5)	3 (18.8)
Asian	0 (0.0)	0 (0.0)
Symptom onset to enrollment, mean (SD), d	8.6 (3.2)	8.6 (3.9)
Body mass index, mean (SD), kg/m^2^	31.9 (9.4)	29.8 (8.0)
<18.5, No. (%)	0 (0.0)	0 (0.0)
18.5-24.9, No. (%)	3 (21.4)	5 (33.3)
25.0-29.9, No. (%)	4 (28.6)	6 (40.0)
≥30, No. (%)	7 (50.0)	4 (26.7)
Acute COVID-19 symptoms, No. (%)		
Cough	14 (87.5)	11 (68.8)
Fatigue	12 (75.0)	11 (68.8)
Fever	11 (68.8)	8 (50.0)
Myalgia	8 (50.0)	9 (56.3)
Joint pain	7 (43.8)	5 (31.3)
Nasal congestion	5 (31.3)	6 (37.5)
Runny nose	4 (25.0)	6 (37.5)
Diarrhea	5 (31.3)	5 (31.3)
Sore throat	5 (31.3)	0 (0.0)
Coexisting diseases, No. (%)		
Hypertension	8 (50.0)	8 (50.0)
Diabetes	6 (37.5)	7 (43.8)
Cardiovascular disease	2 (12.5)	4 (25.0)
Chronic obstructive pulmonar disease	2 (12.5)	3 (18.8)
Asthma	0 (0)	1 (6.3)
Chronic kidney disease	0 (0.0)	0 (0.0)
Rheumatic disease	0 (0.0)	0 (0.0)
Concomitant medications, No. (%)		
Antibiotic	10 (62.5)	13 (81.3)
Anticoagulant	13 (81.3)	9 (56.3)
Corticosteroids	8 (50.0)	7 (43.8)
Antihypertensive	8 (50.0)	7 (43.8)
Proton pump inhibitor	4 (25.0)	7 (43.8)
Analgesic	4 (25.0)	5 (31.3)
Antiemetic	3 (18.8)	5 (31.3)
Hypoglycemic	5 (31.3)	2 (12.5)
Hypolipidemic	2 (12.5)	3 (18.8)
Thyroid	3 (18.8)	1 (6.3)
Antiviral^b^	1 (6.3)	0 (0.0)
Oxygen supplementation, No. (%)		
No oxygen therapy	6 (37.5)	0 (0)
Oxygen therapy	9 (56.3)	15 (93.8)
Non-invasive ventilation	1 (6.3)	1 (6.3)
Computed tomography findings, No. (%)		
Ground-glass opacities 50%	7 (53.8)	5 (50.0)
Ground-glass opacities 50%	6 (46.2)	5 (50.0)
Laboratory variables		
25-hydroxyvitamin D, mean (SD), ng/mL	7.7 (1.6)	7.9 (1.6)
Total calcium, mean (SD), mg/dL	8.4 (0.6)	8.4 (0.5)
Creatinine, mean (SD), mg/dL	0.84 (0.48)	0.86 (0.39)
C-reactive protein, median (IQR), mg/L	70.9 (18.5-134.0)	66.9 (35.0; 141.8)
D-dimer, median (IQR), ng/mL	769 (595-1972)	1385 (836-2283)

a Pardo is the exact term used in Brazilian Portuguese, meaning “mixed ethnicity,” according to the Brazilian Institute of Geography and Statistics.

b The antiviral use involved one patient from the vitamin D_3_ group receiving acyclovir 400 mg bid for herpes zoster prophylaxis.
